# Evaluation of Cell Binding Activities of *Leptospira* ECM Adhesins

**DOI:** 10.1371/journal.pntd.0003712

**Published:** 2015-04-14

**Authors:** Gregory T. Robbins, Beth L. Hahn, Karen V. Evangelista, Lavinia Padmore, Patrick S. Aranda, Jenifer Coburn

**Affiliations:** 1 Medical College of Wisconsin, Milwaukee, Wisconsin, United States of America; 2 Division of Infectious Diseases, Department of Medicine, Medical College of Wisconsin, Milwaukee, Wisconsin, United States of America; 3 Graduate Program in Microbiology, Immunology, and Molecular Genetics, Medical College of Wisconsin, Milwaukee, Wisconsin, United States of America; University of Tennessee, UNITED STATES

## Abstract

Pathogenic spirochetes of the genus *Leptospira* are the causative agents of leptospirosis, a zoonotic infection that occurs globally. The bacteria colonize the renal proximal tubules of many animals and are shed in the urine. Contact with the urine, or with water contaminated with the urine of infected animals can cause infection of new host animals, including humans. Mechanisms of colonization of the proximal tubule and other tissues are not known, but specific interactions between bacterial adhesins and host substrates are likely to be critical in this process. Several extracellular matrix (ECM) adhesins have been previously identified, but more recently, it has been shown that *Leptospira* bind more efficiently to cells than ECM. In this work, recombinant forms of five putative *Leptospira* ECM adhesins, namely LipL32, Loa22, OmpL1, p31/LipL45, and LenA were evaluated for binding to cells as well as an expanded variety of ECM components. Reproducible and significant adhesin activity was demonstrated only for OmpL1, which bound to both mammalian cell lines tested and to glycosaminoglycans (GAGs). While determination of biologically significant bacterial adhesion activity will require generation of site-directed mutant strains, our results suggest that OmpL1 is a strong candidate for future evaluation regarding the roles of the adhesin activity of the protein during *L*. *interrogans* infection.

## Author Summary

Leptospirosis is the most widespread zoonotic infection in the world and represents a major public health problem, especially in tropical climates. The processes by which some *Leptospira* species cause infection, disease, and colonization of carrier animals remains poorly understood. Specific binding of *Leptospira* molecules and host molecules are likely important for infection and colonization. To identify *Leptospira* molecules that mediate attachment to host substrates, prior studies have evaluated *Leptospira* membrane proteins for binding to extracellular matrix (ECM) proteins. More recent data, however, show that *Leptospira* bind more efficiently to cells than to ECM. In search of adhesins mediating the latter activity, our study evaluated the direct cell binding activity of recombinant forms of a group of previously reported *Leptospira* ECM adhesins. Only one of these proteins, OmpL1, demonstrated reproducible direct cell binding activity. Further work will focus on identification of the mammalian receptor for OmpL1 and determining the biological significance of this activity during infection.

## Introduction

Pathogenic species of *Leptospira*, a genus of the Gram-negative, spiral-shaped *Spirochaeta* family, are the causative agents of leptospirosis, a zoonotic infection that occurs globally (reviewed in [1–4]). The incidence is especially high in tropical climates at 10 cases per 100,000 per year, whereas in temperate climates the estimated incidence is only 0.1–1 cases per 100,000 per year [5]. The disease is likely underreported, however, due to the non-specific and varied nature of its symptoms, ranging from mild and non-specific (e.g. fever, headache, arthralgias) to severe disease that can include multi-organ failure [1, 2, 6]. The case fatality rate for severe leptospirosis, also known as Weil’s Disease, can be 5–40% (reviewed in [1–4]).

Leptospirosis is spread through the contact of mucous membranes, cuts, or abrasions with fresh water contaminated by the urine of infected animals, or *via* direct contact with urine from infected animals (reviewed in [1–4]). Pathogenic *Leptospira* have been reported to survive for up to several months in fresh water at a neutral or mildly alkaline pH within a temperature range of 28–32°C [2, 6]. A majority of mammalian species have been shown to be potential carriers for *Leptospira*, including rodents, dogs, cats, cattle, pigs, and horses. Within these reservoir animals, the *Leptospira* colonize the kidneys (specifically the luminal aspect of the proximal tubule epithelium) without causing symptoms, while they slough off continually into the urine (reviewed in [1–4]). Human populations at risk for sporadic cases of leptospirosis include farmers, sewer workers, veterinarians, and slaughterhouse workers, but outbreaks of this disease are often associated with severe flooding events, particularly in tropical regions [6–8].

Prevention of disease in humans is based primarily on controlling the disease in animals. The purpose of vaccinating animals is to protect the individual animals and herds from disease, and to prevent spread to humans. However, there are several problems with the available vaccines, including poor cross-protection against most of the more than 250 serovars of *Leptospira*, a wide range of reported adverse reactions, and lack of protection against carriage by maintenance hosts, which continue shedding the bacteria in the urine, perpetuating contamination of the environment. Several outer surface proteins, some of which are also adhesins, have been investigated as vaccine candidates. It is clear from numerous studies that several promising vaccine candidates are under evaluation [9–18]. The outer membrane porin OmpL1 has shown potential, particularly as a component of a subunit vaccine [11, 13, 19–21]. OmpL1 conferred partial immunity to *Leptospira* in hamsters when combined with LipL41, but not alone, and effectiveness was dependent on delivery in membrane fractions of the recombinant *E*. *coli* engineered to produce the proteins [13]. Although recent work has demonstrated that LipL32 is not primarily surface-exposed [22], LipL32 was shown by one group to confer partial immunity to infection in hamsters when combined with the B subunit of *E*. *coli* heat-labile enterotoxin [23]. However, results from a different group did not show protection after immunization with either LipL32 or LigA [15], illustrating the lack of reproducibility in generation of protective responses that has plagued the field. Consistent with the lack of protective immunity and subsurface localization, LipL32 is not required for infection [24].

Several of the vaccine candidates evaluated to date have also been identified as adhesins that bind to extracellular matrix (ECM) components. Adhesion to the host cells in culture and to the renal proximal tubule epithelial cells *in vivo* is a shared trait among pathogenic *Leptospira* species (as reviewed in [25]), and by definition, physiologically relevant adhesins must be exposed on the bacterial surface during infection. Thus they are among the outer surface proteins that are attractive candidates for vaccine development. Adhesins that have been investigated as candidate vaccines [10, 11, 13, 18] include LigA, which binds to fibronectin, fibrinogen, and more weakly to collagen types I and IV [26], LipL32, which binds fibronectin and collagen type IV [27–29], and OmpL1, which binds to laminin, fibronectin and more efficiently to plasminogen [30].

In the work reported here, we tested recombinant versions of several *L*. *interrogans* ECM adhesins and putative surface proteins for adhesion to a variety of mammalian substrates that we and others have reported (e.g. [30–37]). These substrates included fibronectin, laminin, collagen, GAGs, and human cells in culture. The *L*. *interrogans* proteins tested included OmpL1, LipL32, and LenA (also identified as Lsa24/LfhH). LenA is a member of the leptospiral endostatin-like protein (Len) family, and binds to a large number of host molecules including complement factor H, factor H-related protein-1, plasminogen, laminin, and fibronectin [37, 38]. We also tested select proteins previously shown to localize to the outer membrane, such as LipL48 [39, 40] and p31/45 [41], as well as the known virulence determinant Loa22 [42], for adhesin activity in this work. Although most studies in the field have examined *Leptospira* interactions with components of the ECM or with soluble host proteins, *L*. *interrogans* was recently shown to bind more efficiently to the surface of mammalian cells than to the ECM deposited by the cells [31, 43]. The aim of this study was to determine whether selected leptospiral ECM adhesins (LenA, LipL32, and OmpL1), a known virulence factor (Loa22), and/or surface proteins (p31/45 of LipL48) bind directly to mammalian cells, and to evaluate relative binding activities of the *L*. *interrogans* proteins to different mammalian substrates.

## Materials and Methods

### Cloning of *Leptospira interrogans* Genes

*Leptospira interrogans* serovar Copenhageni (pathogenic, strain Fiocruz L1-130) DNA prepared as previously described [44] was used to amplify the genes encoding the proteins of interest. The portion of each gene encoding the amino terminal protein secretion signal was identified using previously published rules [45, 46], and primers were designed to exclude this region and amplify the portion encoding the mature protein. All primers used for amplification of the selected gene fragments are listed in Table 1. The PCR products were purified using QIAquick (Qiagen, Valencia, CA), then digested with the restriction enzymes according to the sites incorporated into the primers. The digested fragments were ligated to pMalC2 or pMalP2 (New England Biolabs, Beverly, MA) digested with the same enzymes. The ligation mixtures were introduced into *E*. *coli* TOP10 (Invitrogen, Life Technologies, Grand Island, NY) by electroporation, and transformants were selected on plates containing 100 μg/ml ampicillin plus 0.2% w/v dextrose. Single colonies were re-streaked to obtain clones for sequencing (primers listed in Table 1); only those that corresponded to the sequence of the *L*. *interrogans* genes were used for further experimentation. Plasmids containing the correct sequences were introduced into *E*. *coli* KS330 [47] for protein production. MBP fusions were chosen for the work here because they have previously been used to produce other bacterial outer membrane proteins with adhesin activity in soluble form in *E*. *coli* [48, 49], which minimizes potential deleterious effects of denaturation and renaturation.

**Table 1:** Oligonucleotides used in this study.

Name	Sequence^1^, 5’-3’	Purpose	Reference
*lipL32*F	TTCAGAATTCGGTGCTTTCGGTGGTCTG	Cloning	annealing portion from (28)
*lipL32*R	GCAGGTCGACTTACTTAGTCGCGTCAGAAGAC	Cloning	annealing portion from (28)
*loa22*F	TTCAGAATTCGAGGAATCCGCAGCTCC	Cloning	this study
*loa22*R	GCAGGTCGACTTATTGTTGTGGTGCGG	Cloning	annealing portion from (76)
*ompL1*F	TTCAGAATTCAAAACATATGCAATTGTAGGATTTGG	Cloning	this study
*ompL1*R	GCAGGTCGACTTAGAGTTCGTGTTTATAACCGAATCTG	Cloning	this study
*lenAF*	ATTCGGATCCGGGGATAAAAAAGAAGAAGATAATTCAG	Cloning	this study
*lenAR*	TTGCCTGCAGTTACTGTTCTACACAGAGAAGATTTAGATTG	Cloning	this study
*lipL48F*	TTCAGAATTCAACTTATACGTAACTCCTTCCCTCTATC	Cloning	this study
*lipL48R*	GCAGGTCGACTTATCTCGCTCTATAAACGGTCC	Cloning	this study
*p31/45F*	TTCAGAATTCAAGAAACCTACCGAAAGTTCCAAG	Cloning	this study
*P31/45R*	GCAGGTCGACTCAGAATTTAGCTTTTGTTTGGAAG	Cloning	this study
oMal01	CGCTTTCTGGTATGCCGTGCGTA	Sequencing	(48)
oMal02	TCTCATCCGCCAAAACAGCCAAG	Sequencing	(48)
LipL48.f1	TCTCGCTTTGTTAGCGGGAG	Sequencing	this study
LipL48.r1	GCACTTAATGCGGAACCAGC	Sequencing	this study
LipL48.f2	TGGAAATAGAGCTCCGGGCG	Sequencing	this study
LipL48.r2	GCGGTACCACCACAACTGG	Sequencing	this study
LipL48.f3	GACTGGTGGAATGGACGACG	Sequencing	this study
LipL48.r3	GAAGACCGGTTCCACCTTCC	Sequencing	this study
LipL48.f4	TGTAGCCAATGGAGGTGGTG	Sequencing	this study
LipL48.r4	GTTTCCACCTTGAACGGAAGC	Sequencing	this study
LenA.f1	TTTGGAGCAGACGCAAAGTG	Sequencing	this study
LenA.r1	TCCCGTAATAAGTCCGTCCG	Sequencing	this study
LenA.f2	AATACCGTAGAAGCGACGGC	Sequencing	this study
LenA.r2	GACCGTCGACAATCAAAGCC	Sequencing	this study
P31.f1	AAGTTGATATTCAATTTGCAGACGG	Sequencing	this study
P31.r1	TTGACGAGCTGCTACTTCGC	Sequencing	this study
P31.f2	GCAGACCGTTGCTTCTTCAG	Sequencing	this study
P31.r2	ACAATCTCTGAAGAAGCAACGG	Sequencing	this study
P31.f3	GAAAGCGAAGTAGCAGCTCG	Sequencing	this study
P31.r3	GAGATTCGAATTGCAGATCCGTC	Sequencing	this study

^1^Restriction sites used for cloning are underlined.

### Production of Maltose Binding Protein (MBP) Fusion Proteins

Production and purification of MBP-fusion proteins were performed as described previously [44, 48]. Briefly, LB broth supplemented with dextrose to 0.2% and ampicillin to 100 μg/ml was inoculated from glycerol stocks of *E*. *coli* containing the pMalC2 or pMalP2 plasmids and incubated overnight at 30°C with shaking. On the following day, the cultures were diluted 1:100 by adding 5 ml of the cultures to 500 ml 2x YT medium supplemented with benzamidine to 1 mM, dextrose to 0.2%, and ampicillin to 100 μg/ml. These preparations were then incubated at 30°C with shaking and monitored for growth by measuring the optical density at 600 nm. When the OD_600_ reached 0.4–0.6, 5 ml was transferred to a separate tube for use as an uninduced negative control. To the remaining culture, isopropyl-β-D-thiogalactopyranoside (IPTG) and leupeptin were added to final concentrations of 1 mM and 0.01 μg/ml, respectively, and incubation was continued at 30°C for 2 hr with shaking. The induced cultures were then transferred to centrifuge bottles and left on ice for 20 min., then centrifuged at 2,970 x g and 4°C for 20 min. The supernatants were discarded and the pellets washed with 25mM HEPES pH 7.8, 1 mM EDTA, 150 mM NaCl, 1 mM benzamidine, and 0.01 TIU/ml aprotinin (HBS+2PI). The final pellets were stored at -80°C until further use. Protein production in samples taken from each stage was assessed by immunoblot using anti-MBP (New England Biolabs).

Recombinant proteins were released from the pelleted *E*. *coli* cells using a French Pressure cell. The buffer used throughout was HBS+2PI, with the addition of PMSF to 1 μM immediately before cell breakage. The broken cell suspensions were centrifuged at 26,650 x g for 30 min at 4°C, and the pellets and supernatants assessed for the presence of recombinant protein. Recombinant proteins were purified from the supernatants using amylose affinity chromatography as previously described [44, 48]. The concentrations of the purified proteins were measured using the Bio-Rad Protein Assay (Bio-Rad, Hercules, CA), and the stocks were stored at -80°C until use in adhesion assays. A stained gel and an immunoblot of the protein preparations are shown in S1 Fig All migrated at the expected relative mobilities based on molecular weights.

### Adhesion Assays

To screen for ECM molecule recognition by recombinant by *L*. *interrogans* adhesins, varying concentrations of substrates were initially tested. Optimal coating concentrations identified from the preliminary studies were 0.03 μM for fibronectin (from human plasma #F0895, Sigma-Aldrich, St. Louis, MO), laminin (#L2020, Sigma-Aldrich), and type IV collagen (both purified from Engelbreth-Holm-Swarm murine sarcoma basement membrane, #C0543, Sigma-Aldrich), and 1 mg/ml GAGs chondroitin sulfates A (#C9819, Sigma-Aldrich), B (#C3788, Sigma-Aldrich), and C (#C4384, Sigma-Aldrich), heparin (#01491, Polysciences, Inc., Warrington, PA) and heparan sulfate (#H7640, Sigma-Aldrich). All were solubilized in HBS (25 mM HEPES pH 7.4, 150 mM NaCl). Fifty μl/well of each purified substrate was added to non-tissue culture 96-well plates immediately after the coating solution was made. In parallel, the vehicle (HBS) with no added protein was plated. The plates were incubated at 4°C overnight, then washed with HBS 3 times with 200 μl/well (3 x 200 μl) and blocked for 1 hr at room temperature (RT) in a solution of HBSB (50 mM HEPES, 150 mM NaCl, 1 mM MgCl_2_, 1% bovine serum albumin (BSA)) essentially as previously described [31]. The blocking solution was siphoned off, and the recombinant proteins at 1 μM (determined in preliminary experiments) in HBSB were added to the wells. Following incubation at RT for 1 hr on a platform shaker, the wells were washed 3 x 200 μl HBS, incubated 5 min at RT on the shaker, washed once more, and then fixed with 3% paraformaldehyde in PBS for 30 min. The wells were then blocked 1 hr RT with 150 μl 1 x TBS containing 1% BSA and 3% normal goat serum at 4°C. Binding of all fusion proteins was quantified by ELISA using anti-MBP rabbit antiserum (New England Biolabs) diluted 1:10,000, followed by either goat anti-rabbit IgG conjugated to alkaline phosphatase (1:10,000) or anti-rabbit IgG conjugated to HRP (1:10,000). AP and HRP activities were detected using commercially available colorimetric substrates and the plates read at 405 or 655 nm, respectively, within the linear development range of the assay.

Evaluation of direct cell binding activity of the MBP-adhesins involved two human cell lines. Ea.hy926 macrovascular endothelial cells [50, 51], generously donated by Dr. C.J. Edgell from UNC, Chapel Hill, were used at passage ≤ 6 from Dr. Edgell’s laboratory. HEp2 laryngeal carcinoma cells were purchased from ATCC. Both cell lines were cultured under the conditions recommended by the source. The cell binding assays were performed in 48 well plates. The cells were seeded 1–3 days before the cell binding assay to achieve confluence or post-confluence on the day of the binding assay. The medium was removed, and the cells were washed once with 500 μl HBSC +2PI (25 mM HEPES pH 7.4, 150 mM NaCl, 1 mM MgCl_2_, 1 mM MnCl_2_, 0.25 mM CaCl_2_ supplemented with 10^-2^ TIU/ml aprotinin and 1 mM benzamidine) before the addition of recombinant proteins at 0.1 μM in serum-free (Dulbecco’s modified Eagle medium (DMEM; Gibco, Grand Island, NY), as optimized in preliminary experiments. The cells were incubated with the proteins for 1 hour at 37°C, followed by removal of medium, washing 3 times with 500 μl HBSC +2PI, and fixing in 150 μl 3% paraformaldehyde in PBS for 30 min at RT. Binding was quantified by ELISA as described above.

In all experiments, binding of MBP-adhesin fusions to the cells or purified substrates were compared to binding of the proteins to wells in which vehicle alone had been plated. The binding activity of the MBP-adhesin fusion constructs was also compared to negative and positive controls. The negative controls included medium without added recombinant protein, and the MBP-β-gal fusion produced by the empty vector. As a positive control for cell binding an MBP fusion to an integrin-binding fragment of invasin from *Yersinia pseudotuberculosis* was used.

### Analysis of Adhesion Data

**Fig 1:**
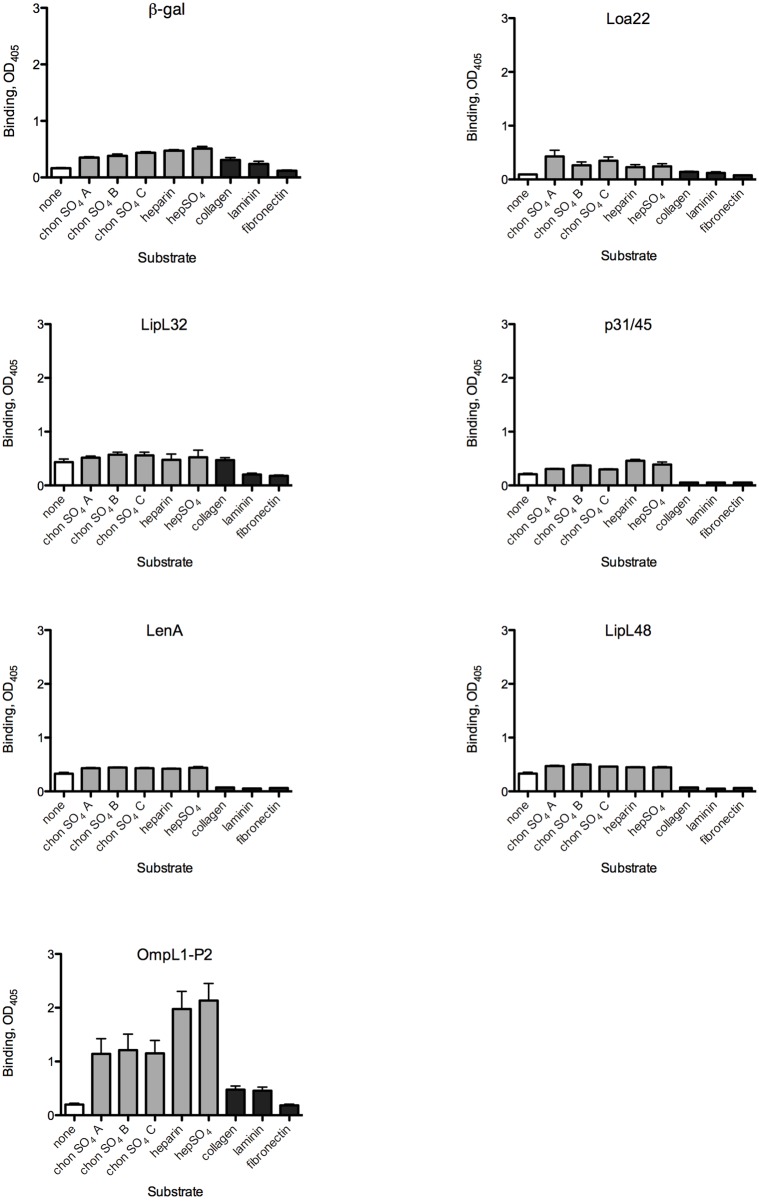
Evaluation of candidate *L*. *interrogans* adhesin attachment to mammalian substrates. MBP fusions to *L*. *interrogans* proteins were used to probe wells coated with the substrate molecules as described in Materials and Methods. After removal of unbound proteins, binding was quantified by ELISA using an anti-MBP antibody. Abbreviations: chonSO_4_ = chondroitin sulfate, hepSO_4_ = heparan sulfate. Bars indicate means + standard errors; n = 4–28 replicates. White bars: buffer control; gray bars: glycosaminoglycans; black bars: proteins. Statistical analyses for all comparisons are provided in S1 Table.

The binding activity of each protein is presented after subtraction of the assay background, i.e. the OD value of wells containing no substrate and no recombinant adhesin protein. The inherent “stickiness” of each recombinant candidate adhesin was determined using wells to which no purified substrate was added after subtraction of the assay background, and is shown in each case (Fig 1). For cell binding assays, wells in which the culture medium with no cells had been plated were used to subtract background binding to medium components, and the means ± SEM of the background-subtracted data are presented in the figures. To estimate *K*_D_s, the xy analysis subprogram in GraphPad Prism v. 5 was used. Statistical analyses were performed using parametric one-way ANOVA followed by Tukey’s multiple comparisons test in GraphPad Prism v. 5. A *P*-value of ≤ 0.05 was considered to be statistically significant.

## Results

The abilities of six MBP fusions to *L*. *interrogans* proteins with reported ECM binding activities and/or outer surface exposure were evaluated by expressing the mature coding sequences as fusions to maltose-binding protein. The vector-encoded fusion to the α fragment of β-galactosidase (β-gal) was purified in parallel with the recombinant *L*. *interrogans* adhesins and used as the background control protein in all studies presented here. Each MBP-fusion protein was initially tested for the ability to bind to eight purified molecules that were previously shown to bind *L*. *interrogans* and that are components of the extracellular matrix. Some are also cell-associated, e.g. GAGs that are covalently linked to mammalian cell membrane proteins to form proteoglycans. Attachment to wells containing the buffer control as well as the eight substrates is shown in Fig 1. Statistical analyses are presented in S1 Table. None of the recombinant proteins showed increased attachment to collagen, laminin, or fibronectin as compared to the buffer control, despite the abilities of all three substrate proteins to bind intact *L*. *interrogans* (Fig 1 and [43]). In fact, for several of the MBP fusion proteins, binding to the collagen, laminin, and fibronectin-containing wells was significantly less efficient (P ≤ 0.05) than was binding to the buffer control, suggesting that these ECM proteins might occupy non-specific binding sites on the plastic surface that bind to the candidate adhesins despite blocking with BSA. This was the case for the MBP fusions to LipL32 in binding to fibronectin and collagen-coated wells, and for MBP-p31/45,-LenA, and-LipL48 binding to all three ECM proteins. Over a range of protein concentrations, OmpL1 did show some binding to fibronectin (*K*_D_ ≈ 0.87 ± 0.5 μM), which is similar to the previously published *K*_D_ for OmpL1 binding to fibronectin (≈ 1.2 ± 0.5 μM [30]). Other *L*. *interrogans*-derived MBP fusion proteins and MBP-β–gal did not (Fig 1). Thus, although LipL32 and LenA have been reported to bind to ECM proteins in other contexts [33, 37], the results are not reproducible when MBP fusions are used.

**Fig 2:**
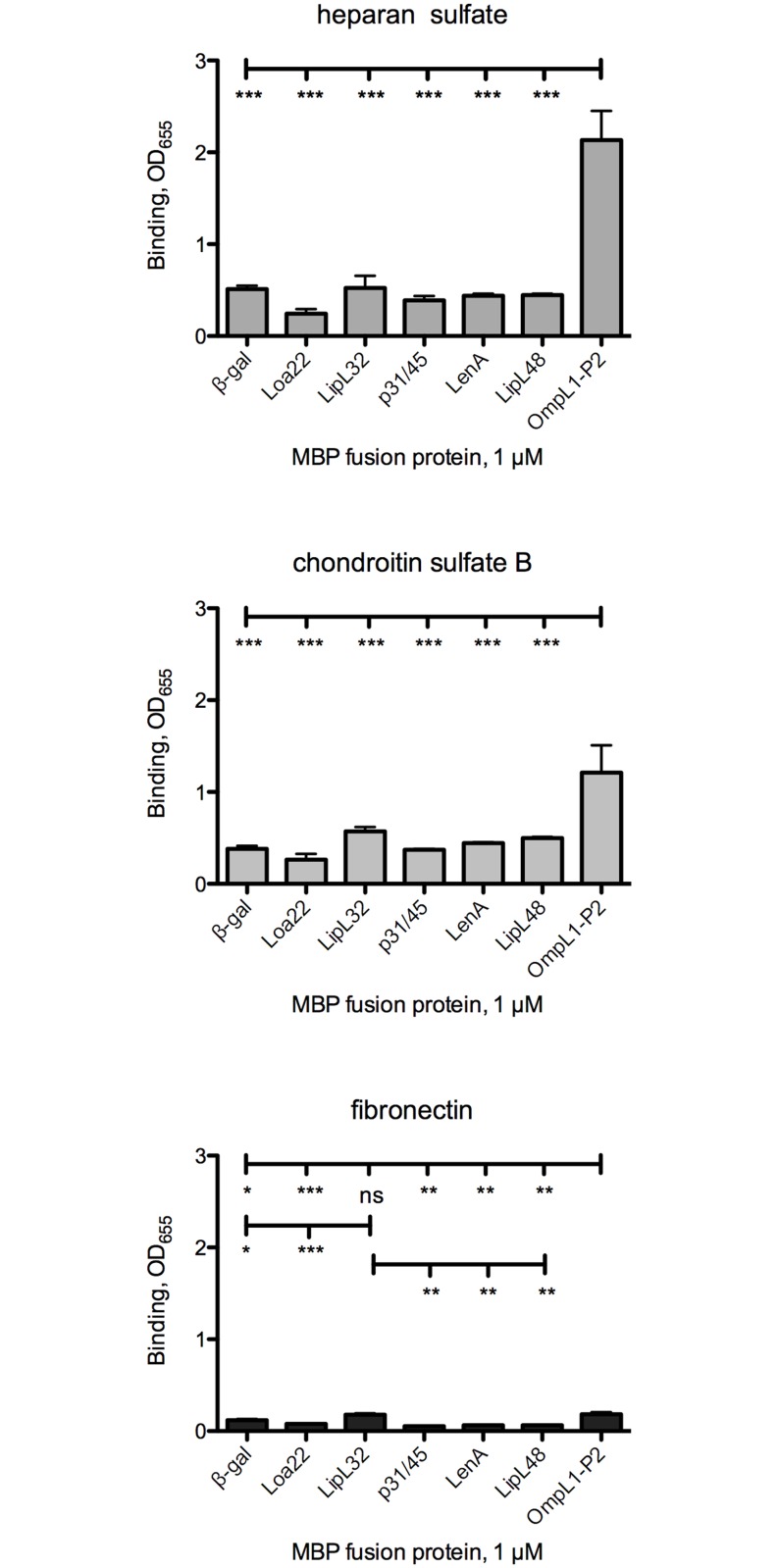
Evaluation of select host molecules as targets for *L*. *interrogans* candidate adhesin attachment. Data obtained in the experiments shown in Fig 1 were re-graphed to highlight candidate adhesin binding to substrates previously demonstrated to bind intact bacteria. Bars indicate means + standard error; n = 4–28 replicates. Statistical analyses for all comparisons are provided in S2 Table.

Testing of proteoglycans as potential substrates for *L*. *interrogans* protein adhesion did reveal some statistically significant binding as compared to the buffer control wells. LipL48 and OmpL1 bound significantly more efficiently to all 5 proteoglycans tested than to the buffer control, while Loa22, p31/45, and LenA, bound to some of the proteoglycans (Fig 1). The caveat to most of these results was that the MBP-β-gal also bound more efficiently to the proteoglycans, raising doubts regarding the biological relevance of the results with the candidate adhesins. To compare the binding efficiencies of the recombinant MBP fusions to select substrates that are known to bind *L*. *interrogans* [31, 52, 53], we re-analyzed the same data set. This allowed determination of whether any of the recombinant fusions to *L*. *interrogans* proteins bound significantly more efficiently to any substrate than did the fusion to β-gal (Fig 2; statistical analyses are presented in S2 Table). These analyses revealed that MBP-OmpL1 bound significantly more efficiently than did any of the other MBP fusions (including β-gal) to heparan sulfate and chondroitin sulfate B, both of which are substrates for *L*. *interrogans* attachment. The estimated *K*_D_ for OmpL1 binding to heparan sulfate (≈ 0.16 + 0.08 μM) showed higher affinity binding to heparan sulfate than to fibronectin, while a *K*_D_ for MBP-β-gal could not be estimated due to weaker binding. As described above, MBP-OmpL1 also bound to fibronectin significantly more than did β-gal, Loa22, p31/45, LenA, and LipL48. Similarly, LipL32 bound to fibronectin at low levels but significantly more efficiently than did β-gal, Loa22, p31/45, LenA, and LipL48. Thus, of the six *L*. *interrogans* proteins tested for adhesin activity, only OmpL1 and LipL32 showed any significant binding above the background of the control protein MBP-β-gal to any of the eight substrates tested. Since LipL32 was recently shown to be primarily a subsurface protein [22], OmpL1 is the only candidate tested that has significant adhesin activity that has the potential for biological relevance.

**Fig 3:**
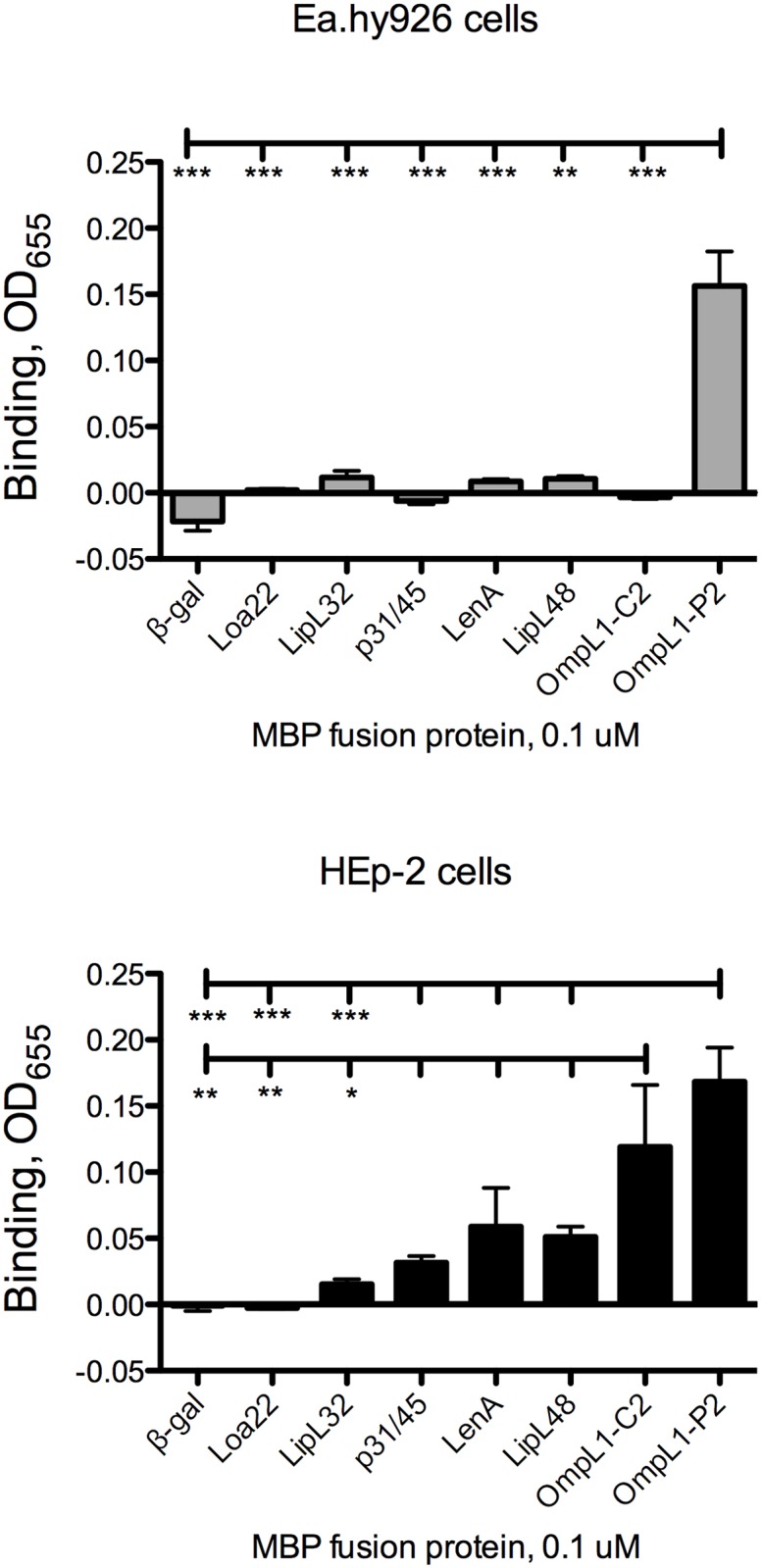
Evaluation of candidate *L*. *interrogans* adhesin attachment to human cell layers. Confluent cell layers were probed with MBP fusions to candidate *L*. *interrogans* adhesins as described in Materials and Methods. Bars indicate means + standard error; n = 4–24 replicates. Statistical analyses for all comparisons are provided in S3 Table. * = *P*<0.05; ** = *P*<0.01; *** = *P*< 0.001.

*L*. *interrogans* has been shown to bind to mammalian cells in culture by a number of laboratories, and intact cell layers would potentially present a number of potential receptors available for *L*. *interrogans* attachment. These receptors would also be in native conformation, a concern when purified substrates are immobilized in plastic wells. We therefore tested all six recombinant *L*. *interrogans* proteins and MBP-β-gal for the ability to bind to a human endothelial cell line and to a human epithelial cell line. MBP-OmpL1 was the only *L*. *interrogans* protein that bound to the endothelial cell line Ea.hy926 above the background level of MBP-β-gal (Fig 3; statistical analyses are presented in S3 Table). The context of fusion protein expression, however, significantly impacted the binding activity of OmpL1 to the endothelial cell line, as expression of the fusion protein that is retained in the cytoplasm (gene cloned in pMalC2) had no significant binding activity as compared to MBP-β-gal, while the MBP fusion protein exported to the periplasm (gene cloned in pMalP2) had significantly higher binding activity. In contrast, there was no statistically significant difference between the two versions of MBP-OmpL1 in binding to HEp-2 epithelial cells, and both bound significantly more efficiently to the cells than did the β-gal, Loa22, and LipL32 fusion proteins (Fig 3). Interestingly, although the other *L*. *interrogans* did show some apparent attachment to HEp-2 cells, none bound significantly more efficiently to the cells than did β-gal, or less efficiently than did OmpL1. Together, these data suggest that OmpL1 is the only *L*. *interrogans* protein tested that has significant cell binding activity, and that OmpL1 interactions with endothelial cell surface molecules are likely to be more dependent on subtle conformation differences than is the case for the epithelial cell surface molecules.

## Discussion

A large number of candidate *L*. *interrogans* adhesins have been identified by multiple groups [26, 32, 33, 54–66]. Most candidate adhesins have been evaluated for attachment to ECM molecules and soluble host proteins, but only a few have been tested for cell-binding activity. Many of the mammalian ECM and soluble proteins tested are inherently “sticky” and interact with numerous other mammalian molecules, leading to concerns regarding the biological relevance of these interactions. Determination of biological relevance ultimately will require generation of targeted deletions of the genes encoding the candidate adhesins in the pathogenic leptospires, but given the large number identified to date and the difficulties of generating targeted mutants in *L*. *interrogans*, this presents a formidable challenge. One approach to refining the list of candidate adhesins on which to focus future studies is to test for adhesin activity using different versions of the recombinant fusion protein, replication of attachment activity by independent laboratories, and testing multiple substrates in parallel, along with appropriate controls. In previous work, we evaluated cell-binding adhesins that we recently identified [44], two of which bind to cadherins, for attachment to the purified substrates tested here, and none bound to any of the substrates above background levels.

In this work, only the outer membrane protein OmpL1 was reproducibly found to display significant adhesin activity. This was applicable to the proteoglycans tested and to intact monolayers of human cells. In all cases, not only were the differences between OmpL1 and the control β-gal statistically significant, they also generated signal to noise ratios of ≥ 2.5 in comparisons to the “background” binding of β-gal to heparan sulfate, chondroitin sulfate B, and to the endothelial and epithelial cell lines tested. While this ratio is set arbitrarily, we have applied this in the past as a cutoff in studies of the *B*. *burgdorferi* adhesin P66, which was subsequently shown to be required for murine infection [48, 67]. Interestingly, both OmpL1 and P66 have been identified as outer membrane porins as well as adhesins [19, 68–70].

Although OmpL1 was previously reported to bind to laminin and fibronectin [30], our results suggest that OmpL1 may have a more significant role in binding to GAGs and perhaps additional receptors on mammalian cells. In fact, binding appears to occur independently of fibronectin, as fibronectin is not produced by the HEp-2 cells used in these experiments [71, 72], and OmpL1 did not bind to purified fibronectin at ≥ 2.5 fold above the background binding of the control β-gal in our system.

The other candidate *L*. *interrogans* adhesins tested here bound very modestly (in general the signal to noise ratios were < 2 in binding to purified substrates vs. the buffer control), and only LipL32 showed statistically significant binding to any substrate in comparison to the β-gal control. However, while LipL32 was originally thought to be a *L*. *interrogans* surface protein [29, 33, 73], more recent work done using multiple approaches does not support surface-exposure of this protein [22]. The latter result agrees with the lack of a virulence phenotype of a *lipL32* knockout strain [24]. It is entirely possible, however, that the lack of robust adhesion activity we observed for LipL32, LenA, and OmpL1 (to fibronectin in particular) is due to the use of the relatively large MBP fusion tag masking critical domains of the *L*. *interrogans* proteins, or that the tag alters the conformations of the proteins. This question awaits the analysis of the native proteins purified from *L*. *interrogans*, which are not easily obtainable. The data obtained for the two OmpL1 fusions, however, suggests the possibility that the conformation of the protein is affected by passage through the export pathway.

A number of additional differences in approach are apparent in the different studies of *L*. *interrogans* adhesins. For example, work by Stevenson et al. [37], Barbosa et al. [55], and Verma et al. [38, 74] has collectively shown that LenA binds to factor H and related complement regulatory proteins, laminin, plasminogen, and fibronectin. However, it should be noted that the work by Barbosa is the only study that includes analysis of additional *L*. *interrogans* as well as additional substrates that permit comparisons to be made and specificity of interactions to be evaluated. In future studies it will potentially be worthwhile to further explore the possibility that LenA might bind to additional molecules that have not yet been tested, and that differences in the fibronectin preparation, the fusion tags for the *L*. *interrogans* proteins, and the specific conditions used to assess interactions may be critical. The biological relevance of LipL32 binding as assessed using recombinant proteins *in vitro* has been called into question by a recent re-evaluation of the localization of the protein in the bacterial cell [22].

Adhesion studies *in vitro* are fraught with possibilities of generating artifactual and/or biologically insignificant results. Determination of biologically significant bacterial adhesion activity will require generation of site-directed mutant strains, gain of function strains, identification of the domains and/or amino acids of the adhesins that are responsible for binding activity, and testing of mutant strains in animal models of infection. However, adhesins serve as attractive vaccine candidates because the antibody response may block adhesion activity as well as assist in clearing the bacteria. While P66 does not appear to elicit a protective response in a manner that is achievable outside the research laboratory setting, OmpL1 has shown promise as a component of a subunit vaccine. In addition, the GAG-binding activity of OmpL1 is intriguing, as GAG binding but not fibronectin binding by the *B*. *burgdorferi* adhesin BBK32 was recently shown to mediate tropism to and colonization of joint tissue in mice [75]. These results demonstrated *in vivo* biological relevance of a specific adhesin activity. Together, all of the data cited above, along with the data presented here, suggest that OmpL1 is a strong candidate for future evaluation regarding the roles of the protein, and its adhesion and porin functions, in *L*. *interrogans* infection.

## Supporting Information

S1 Fig Recombinant proteins used in this study. All were generated as fusions to maltose-binding protein (MBP). Panel A shows the Coomassie stain of total protein with 400 ng each MBP fusion loaded per lane. The numbers on the left of each panel show the relative mobilities of the “Broad Range” markers (in KDa) from (New England Biolabs (Beverly, MA, USA). Panel B shows the immunoblot (50 ng each protein) probed with rabbit anti-MBP antiserum (New England Biolabs) diluted 1:10,000. The blot was subsequently probed with anti-rabbit IgG conjugated to alkaline phosphatase (Promega, Madison, WI, USA) followed by colorimetric development. Note that there is some native *E*. *coli* MBP (42.7 kDa) present in each preparation
(PDF)

S1 Table Summary of statistical analyses of comparisons of mammalian substrates bound by candidate *L*. *interrogans* adhesins. Graphical data are presented in Fig 1. * = *P* < 0.05; ** = *P* < 0.01; *** = *P* < 0.001; ns = not significantly different.
(PDF)

S2 Table Summary of statistical analyses of comparisons of *L*. *Interrogans* candidate adhesin binding to mammalian substrates. Graphical data are presented in Fig 2. * = *P* < 0.05; ** = *P* < 0.01; *** = *P* < 0.001; ns = not significantly different.
(PDF)

S3 Table Summary of statistical analyses of comparisons of *L*. *Interrogans* candidate adhesin binding to mammalian cell layers. Graphical data are presented in Fig 3. * = *P* < 0.05; ** = *P* < 0.01; *** = *P* < 0.001; ns = not significantly different.
(PDF)
