# Supplementary material for: Evaluation of Cell Binding Activities of Leptospira ECM Adhesins
Source: PLoS Negl Trop Dis. 2015 Apr 14;9(4):e0003712. doi: 10.1371/journal.pntd.0003712 (PMC4397020; doi:10.1371/journal.pntd.0003712)
Supplement: S1 Fig — All were generated as fusions to maltose-binding protein (MBP). Panel A shows the Coomassie stain of total protein with 400 ng each MBP fusion loaded per lane. The numbers on the left of each panel show the relative mobilities of the “Broad Range” markers (in KDa) from (New England Biolabs (Beverly, MA, USA). Panel B shows the immunoblot (50 ng each protein) probed with rabbit anti-MBP antiserum (New England Biolabs) diluted 1:10,000. The blot was subsequently probed with anti-rabbit IgG conjugated to alkaline phosphatase (Promega, Madison, WI, USA) followed by colorimetric development. Note that there is some native E. coli MBP (42.7 kDa) present in each preparation (PDF) [file pntd.0003712.s001.pdf]

S1 Figure

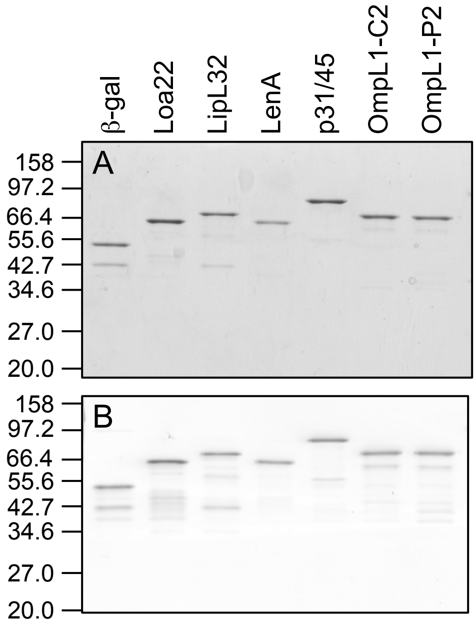

S1 Fig: Recombinant proteins used in this study. All were generated as fusions to maltose-binding protein (MBP). Panel A shows the Coomassie stain of total protein with 400 ng each MBP fusion loaded per lane. The numbers on the left of each panel show the relative mobilities of the "Broad Range" markers (in KDa) from (New England Biolabs (Beverly, MA, USA). Panel B shows the immunoblot (50 ng each protein) probed with rabbit anti-MBP antiserum (New England Biolabs) diluted 1:10,000. The blot was subsequently probed with anti-rabbit IgG conjugated to alkaline phosphatase (Promega, Madison, WI, USA) followed by colorimetric development. Note that there is some native *E. coli* MBP (42.7 kDa) present in each preparation.
