# Supplementary material for: Evaluation of Cell Binding Activities of Leptospira ECM Adhesins
Source: PLoS Negl Trop Dis. 2015 Apr 14;9(4):e0003712. doi: 10.1371/journal.pntd.0003712 (PMC4397020; doi:10.1371/journal.pntd.0003712)
Supplement: S1 Table — Graphical data are presented in Fig 1. * = P < 0.05; ** = P < 0.01; *** = P < 0.001; ns = not significantly different. (PDF) [file pntd.0003712.s002.pdf]

Table S1

SUBSTRATE COMPARISON	β-gal	Loa22	LipL32	p31/45	LenA	LipL48	OmpL1-P2
none vs chon SO_4 A	**	***	ns	*	ns	**	***
none vs chon SO_4 B	***	*	ns	***	*	***	***
none vs chon SO_4 C	***	***	ns	ns	ns	**	***
none vs heparin	***	ns	ns	***	ns	*	***
none vs hepSO_4	***	ns	ns	***	*	*	***
none vs collagen	**	ns	ns	**	***	***	ns
none vs laminin	ns	ns	*	**	***	***	ns
none vs fibronectin	ns	ns	*	**	***	***	ns
chon SO_4 A vs chon SO_4 B	ns	ns	ns	ns	ns	ns	ns
chon SO_4 A vs chon SO_4 C	ns	ns	ns	ns	ns	ns	ns
chon SO_4 A vs heparin	ns	*	ns	**	ns	ns	*
chon SO_4 A vs hepSO_4	ns	ns	ns	ns	ns	ns	**
chon SO_4 A vs collagen	ns	***	ns	***	***	***	ns
chon SO_4 A vs laminin	ns	***	*	***	***	***	*
chon SO_4 A vs fibronectin	***	***	**	***	***	***	***
chon SO_4 B vs chon SO_4 C	ns	ns	ns	ns	ns	ns	ns
chon SO_4 B vs heparin	ns	ns	ns	ns	ns	ns	ns
chon SO_4 B vs hepSO_4	ns	ns	ns	ns	ns	ns	*
chon SO_4 B vs collagen	ns	ns	ns	***	***	***	*
chon SO_4 B vs laminin	ns	ns	**	***	***	***	*
chon SO_4 B vs fibronectin	***	*	***	***	***	***	***
chon SO_4 C vs heparin	ns	ns	ns	**	ns	ns	*
chon SO_4 C vs hepSO_4	ns	ns	ns	ns	ns	ns	**
chon SO_4 C vs collagen	ns	**	ns	***	***	***	ns
chon SO_4 C vs laminin	**	***	**	***	***	***	*
chon SO_4 C vs fibronectin	***	***	**	***	***	***	***
heparin vs hepSO_4	ns	ns	ns	ns	ns	ns	ns
heparin vs collagen	*	ns	ns	***	***	***	***
heparin vs laminin	***	ns	ns	***	***	***	***
heparin vs fibronectin	***	ns	*	***	***	***	***
hepSO_4 vs collagen	**	ns	ns	***	***	***	***
hepSO_4 vs laminin	***	ns	*	***	***	***	***
hepSO_4 vs fibronectin	***	ns	**	***	***	***	***
collagen vs laminin	ns	ns	ns	ns	ns	ns	ns
collagen vs fibronectin	***	ns	*	ns	ns	ns	ns
laminin vs fibronectin	ns	ns	ns	ns	ns	ns	ns
