# Supplementary material for: Evaluation of Cell Binding Activities of Leptospira ECM Adhesins
Source: PLoS Negl Trop Dis. 2015 Apr 14;9(4):e0003712. doi: 10.1371/journal.pntd.0003712 (PMC4397020; doi:10.1371/journal.pntd.0003712)
Supplement: S3 Table — Graphical data are presented in Fig 3. * = P < 0.05; ** = P < 0.01; *** = P < 0.001; ns = not significantly different. (PDF) [file pntd.0003712.s004.pdf]

Table S3

ADHESIN COMPARISON	Ea.hy926 cells	Hep-2 cells
β-gal vs Loa22	ns	ns
β-gal vs LipL32	ns	ns
β-gal vs p31/45	ns	ns
β-gal vs LenA	ns	ns
β-gal vs LipL48	ns	ns
β-gal vs OmpL1-C2	ns	**
β-gal vs OmpL1-P2	***	***
Loa22 vs LipL32	ns	ns
Loa22 vs p31/45	ns	ns
Loa22 vs LenA	ns	ns
Loa22 vs LipL48	ns	ns
Loa22 vs OmpL1-C2	ns	**
Loa22 vs OmpL1-P2	***	***
LipL32 vs p31/45	ns	ns
LipL32 vs LenA	ns	ns
LipL32 vs LipL48	ns	ns
LipL32 vs OmpL1-C2	ns	*
LipL32 vs OmpL1-P2	***	***
p31/45 vs LenA	ns	ns
p31/45 vs LipL48	ns	ns
p31/45 vs OmpL1-C2	ns	ns
p31/45 vs OmpL1-P2	***	ns
LenA vs LipL48	ns	ns
LenA vs OmpL1-C2	ns	ns
LenA vs OmpL1-P2	***	ns
LipL48 vs OmpL1-C2	ns	ns
LipL48 vs OmpL1-P2	**	ns
OmpL1-C2 vs OmpL1-P2	***	ns
